# Supplementary material for: Efficacy of approach bias modification as an add-on to smoking cessation treatment: study protocol for a randomized-controlled double-blind trial
Source: Trials. 2022 Mar 21;23:223. doi: 10.1186/s13063-022-06155-6 (PMC8935694; doi:10.1186/s13063-022-06155-6)
Supplement: Supplementary file 2 — Additional file 2: Table S1. Counterbalancing of Instructions (AAT, IAT) and Order of IATs. Table S2. Ratings of Smoking-Related and Positive Pictures for Each Picture Set Used in the Approach-Avoidance Task (AAT). Table S3. Ratings of Smoking-Related and Positive Pictures Used in the Approach-Avoidance Task (AAT). Table S4. Design of the Single Target Implicit Association Test (ST-IAT) for the Order Compatible-Incompatible. Table S5. Design of the Single Target Implicit Association Test (ST-IAT) for the Order Incompatible-Compatible. Table S6. Picture Numbers (IAPS) of all Control Stimuli. [file 13063_2022_6155_MOESM2_ESM.docx]

Supplementary Online Materials

**Efficacy of Approach Bias Modification as an Add-on to Smoking Cessation Treatment: Study Protocol of a Randomized-Controlled Double-Blind Trial**

Charlotte E. Wittekind^a^*, Keisuke Takano^a^, Philipp Sckopke^a^, Markus H. Winkler^b^, Gabriela G. Werner^a^, Thomas Ehring^a^, Tobias Rüther^c^

^a^ LMU Munich, Department of Psychology, Leopoldstraße 13, 80802 Munich, Germany

^b^ University of Würzburg, Department of Psychology I, Biological Psychology, Clinical Psychology, and Psychotherapy

Address: Marcusstr. 9-11, 97070 Würzburg, Germany

^c^ LMU Munich, University Hospital, Department of Psychiatry and Psychotherapy, Nußbaumstr. 7, 80336 Munich, Germany

***** Corresponding author: Charlotte E. Wittekind, Department of Psychology, LMU Munich, Leopoldstraße 13, 80802 Munich, Germany, charlotte.wittekind@psy.lmu.de, +49 (0)89 2180 5196.

**Supplementary Material 1:**

**Methods**

**Experimental Tasks**

*Counterbalancing*

**Table S1.** Counterbalancing of Instructions (AAT, IAT) and Order of IATs.

| Participant | Order of instruction (AAT) | Order of ST-IATs | Order of instruction (ST-IATs) |
| --- | --- | --- | --- |
| 1 | pull, push | approach-avoid, valence | compatible, incompatible |
| 2 | push, pull | approach-avoid, valence | compatible, incompatible |
| 3 | pull, push | valence, approach-avoid | compatible, incompatible |
| 4 | push, pull | valence, approach-avoid | compatible, incompatible |
| 5 | pull, push | approach-avoid, valence | incompatible - compatible |
| 6 | push, pull | approach-avoid, valence | incompatible - compatible |
| 7 | pull, push | valence, approach-avoid | incompatible - compatible |
| 8 | push, pull | valence, approach-avoid | incompatible - compatible |

*Note.* After eight participants the depicted sequence is repeated. AAT = Approach-Avoidance Task. ST-IAT = Single Target Implicit Association Test.

*Approach-Avoidance Task: Rating*

A sample of 33 adult smokers (age: *M* = 29.36 [range: 18-56]; sex: 64% female; FTND: *M* = 3.21 [range: 0-9]; cigarettes per day: *M* = 11.18 [range: 2-30]) rated all stimuli in an online survey conducted via Unipark^®^. Results are provided in Table S2.

**Table S2.** Ratings of Smoking-Related and Positive Pictures for Each Picture Set Used in the Approach-Avoidance Task (AAT).

| Picture Type | Set 1 | Set 2 | Statistics |
| --- | --- | --- | --- |
| *Craving*  Smoking | 4.24 (2.49) | 4.22 (2.44) | *p* = .682 |
| Positive | 2.26 (1.30) | 2.24 (1.51) | *p* = .798 |
| *Valence*  Smoking | 3.83 (1.81) | 3.82 (1.73) | *p* = .896 |
| Positive | 6.34 (1.43) | 6.37 (1.47) | *p* = .707 |
| *Arousal*  Smoking | 2.56 (2.22) | 2.55 (2.09) | *p* = .968 |
| Positive | 2.29 (1.80) | 2.16 (1.75) | *p* = .179 |

*Note.* ^a^ How strong is your desire to smoke when looking at this picture? (0=not strong at all to 10=very strong); ^b^ How pleasant do you find this picture? (0=no pleasant at all to 10=very pleasant); ^c^ How arousing do you find this picture? (0=not arousing at all to 10=very arousing).

**Table S3.** Ratings of Smoking-Related and Positive Pictures Used in the Approach-Avoidance Task (AAT).

| Picture Type | Smoking | Positive | Statistics |
| --- | --- | --- | --- |
| Craving^a^ | 4.23 (2.46) | 2.25 (1.38) | ***p* < .000** |
| Valence^b^ | 3.82 (1.76) | 6.36 (1.43) | ***p* < .000** |
| Arousal^c^ | 2.55 (2.14) | 2.23 (1.75) | *p* = .157 |

*Note.* ^a^ How strong is your desire to smoke when looking at this picture? (0=not strong at all to 10=very strong); ^b^ How pleasant do you find this picture? (0=no pleasant at all to 10=very pleasant); ^c^ How arousing do you find this picture? (0=not arousing at all to 10=very arousing).

*Single Target Implicit Association Test (ST-IAT)*

*Target stimuli*: butt (Kippe), nicotine (Nikotin), smoking (Rauchen), package (Schachtel), tobacco (Tabak), cigarette (Zigarette)

*Attribute categories approach*. grasp (anfassen), approach (annähern), touch (berühren), reach out (greifen), pull (heranziehen), take (nehmen)

*Attribute categories avoidance*. turn away (abwenden), elude (ausweichen), remove (entfernen), avoid (vermeiden), walk off (weggehen), push away (wegschieben)

*Attribute categories positive valence*. attractive (attraktiv), relaxed (entspannt), sociable (gesellig), happy (glücklich), sexy (sexy), confident (souverän)

*Attribute categories negative valence*. angry (ärgerlich), depressed (depressiv), dangerous (gefährlich), mean (gemein), sick (krank), unhappy (traurig)

There were two different IAT versions: half of the participants first completed the combined compatible blocks (i.e., smoking and approach share one key) and then the combined incompatible block (i.e., smoking and avoidance), whereas the other half received the reversed order (i.e., incompatible-compatible) such that blocks 1, 3, and 4 were switched with blocks 5, 6, and 7 (see Table S4 and S5).

All stimuli were presented centrally on a black screen with labels of the target (German words for “smoking” [Rauchen]) and the attribute categories (German words for approach [Annäherung]/positive [Positiv] and avoidance [Vermeidung]/negative [Negativ]) being presented in either the left or right upright corner of the screen. Presentation of target and attribute stimuli alternated across trials.

**Table S4.** Design of the Single Target Implicit Association Test (ST-IAT) for the Order Compatible-Incompatible.

| **Block** | **No. of trials** | **Items assigned to left key** | **Items assigned to right key** | **Key press**  **left** | **Key press right** |
| --- | --- | --- | --- | --- | --- |
| 1 Attribute practice | 12 | Approach/Positive | Avoid/Negative | 6 | 6 |
| 2 Practice combined block | 24 | Approach/Positive + Smoking | Avoid/Negative | 12 | 12 |
| 3 Test combined block | 48 | Approach/Positive + Smoking | Avoid/Negative | 24 | 24 |
| 4 Practice reversed combined block | 24 | Approach/Positive | Avoid/Negative +  Smoking | 12 | 12 |
| 5 Test reversed combined block | 48 | Approach/Positive | Avoid/Negative +  Smoking | 24 | 24 |

**Table S5.** Design of the Single Target Implicit Association Test (ST-IAT) for the Order Incompatible-Compatible.

| **Block** | **No. of trials** | **Items assigned to left key** | **Items assigned to right key** | **Key press**  **left** | **Key press right** |
| --- | --- | --- | --- | --- | --- |
| 1 Attribute practice | 12 | Approach/Positive | Avoid/Negative | 6 | 6 |
| Practice reversed combined block | 24 | Approach/Positive | Avoid/Negative + Smoking | 12 | 12 |
| Test combined block | 48 | Approach/Positive | Avoid/Negative + Smoking | 24 | 24 |
| Practice combined block | 24 | Approach/Positive +  Smoking | Avoid/Negative | 12 | 12 |
| Test combined block | 48 | Approach/Positive +  Smoking | Avoid/Negative | 24 | 24 |

*Passive Picture Viewing Task and Psychophysiology: Stimuli*

**Table S6.** Picture Numbers (IAPS) of all Control Stimuli.

| Negative | Neutral | Positive |
| --- | --- | --- |
| 1050 | 5500 | 4611 |
| 1120 | 5520 | 4659 |
| 1300 | 5531 | 4670 |
| 1930 | 5532 | 4680 |
| 3010 | 5533 | 4690 |
| 3120 | 5534 | 4695 |
| 3150 | 7000 | 5621 |
| 3170 | 7002 | 8030 |
| 3530 | 7010 | 8190 |
| 6313 | 7025 | 8210 |
| 6350 | 7050 | 8370 |
| 6560 | 7150 | 8490 |

*Post-Intervention Questionnaire Pertaining to the Training and Abstinence*

At the post-intervention assessment, participants are asked to answer the following questions:

1. What do you think, which training did you receive?

- Specific training
- General Training
- No add-on training

1. How do you think does the training work? (open answer)
2. Which positive image that fits to your life as a non-smoker did you pick?
3. Have you used any other smoking-cessation method since the first assessment (e.g., e-cigarettes, nicotine patches, pharmacotherapy)?

- Yes, namely: _______________
- No

1. Have you been smoking since the quit attempt during the smoke-free intervention?

- No, not a single puff
- Yes, 1-5 cigarettes
- Yes, more than 5 cigarettes
